# A Case of Overlapping Extrapyramidal Side Effects and Neuroleptic Malignant Syndrome

**DOI:** 10.1192/bjo.2024.683

**Published:** 2024-08-01

**Authors:** Jun Hua Phan, Kainechukwu Ugwu, Sally Li Er Fong

**Affiliations:** Lincolnshire Partnership NHS Foundation Trust, Lincoln, United Kingdom

## Abstract

**Aims:**

Neuroleptic malignant syndrome (NMS) is a rare, life-threatening idiosyncratic reaction to medications, specifically dopamine receptor antagonists. We report a case of a patient who initially developed extrapyramidal side effects (EPSE) and subsequently developed NMS after being treated with flupentixol depot.

**Methods:**

A 64-year-old woman with an underlying recurrent depressive disorder with psychotic symptoms presented to a psychiatric hospital in June 2023. She exhibited self-neglect, low mood, paranoid delusions, and non-concordance to oral psychiatric medications.

In the first week, she declined all oral medications and was subsequently started on flupentixol decanoate (Depixol) depot injection at 40 mg once every 2 weeks. While showing good improvements in her mental state, she began complaining of akathisia and dystonia since July 2023, consistent with extrapyramidal side effects secondary to flupentixol.

The symptoms improved by lowering flupentixol to 30 mg every 2 weeks and adding procyclidine 5 mg twice daily and propranolol 20 mg three times daily.

In early September 2023, she experienced severe restlessness, stiffness, muscle weakness and felt hot and clammy over 36 hours. Physical observations showed fever, tachycardia, and hypertension. Examination revealed diaphoresis, rigidity in both upper and lower limbs, lower limb weakness, and normal reflexes. Blood tests indicated acute kidney injury (AKI) stage 1, deranged liver function tests, and a creatinine kinase (CK) level of 9405.

She was promptly admitted to the medical hospital for NMS and received extensive intravenous fluid rehydration along with oral Dantrolene. She made a complete recovery, and Depixol was discontinued. Two weeks later, she was started on quetiapine and gradually titrated to 50 mg once daily.

**Results:**

EPSE and NMS are associated with dopamine receptor blockade and commonly occur during the initiation or dosage increment of neuroleptic medications.

NMS is rare but life-threatening, presenting with manifestations of muscle rigidity, pyrexia, altered mental status, sympathetic nervous system lability and elevated CK.

In our case, our patient, who recently started taking neuroleptic medication, experienced EPSE and later deteriorated acutely, raising a high suspicion of NMS. It is essential to consider other possible diagnoses, including serotonin syndrome, malignant hyperthermia, malignant catatonia and electrolyte disturbances.

The commonly used diagnostic criteria include Diagnostic and Statistical Manual of Mental Disorders Fifth Edition (DSM-5) and Levenson's criteria but diagnosis of NMS remains clinical.

The crucial step after identifying NMS is to immediately stop the neuroleptic agent, followed by supportive medical treatment.

**Conclusion:**

Early recognition and prompt treatment of NMS in our patient led to a full recovery.

